# Supplementary material for: T cell-specific deletion of Pgam1 reveals a critical role for glycolysis in T cell responses
Source: Commun Biol. 2020 Jul 24;3:394. doi: 10.1038/s42003-020-01122-w (PMC7382475; doi:10.1038/s42003-020-01122-w)
Supplement: Supplementary file 1 — Supplementary Information [file 42003_2020_1122_MOESM1_ESM.pdf]

Supplementary Figure 1

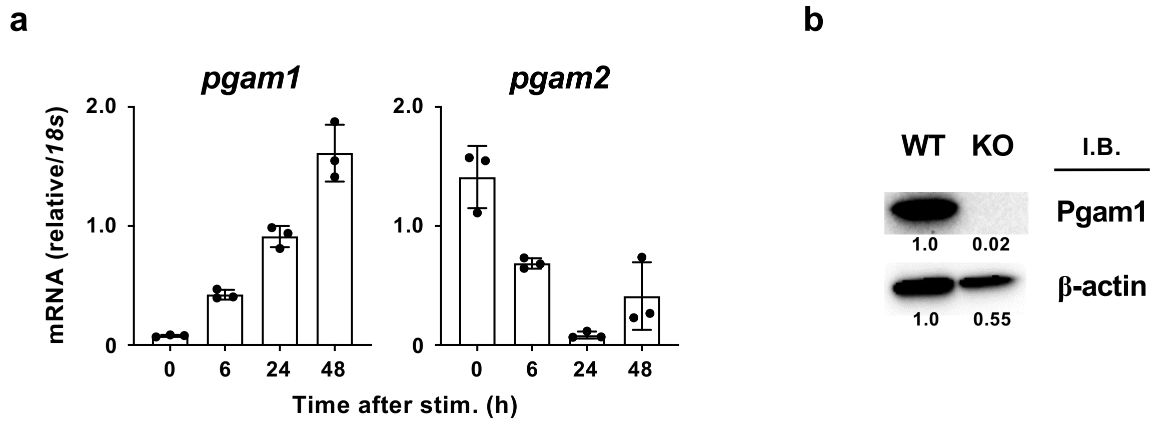

Supplementary Figure 1. (a) Naïve CD8 T cells were stimulated with anti-TCR-β plus anti-CD28 mAbs for the indicated hours and the mRNA expression of *pgam1* and *pgam2* was determined by quantitative RT-PCR. The results are presented relative to the expression of *18s* rRNA with the standard deviations (n = 3, technical replicates). **(b)** Pgam1 and β-actin levels in wild-type and *Pgam1* KO CD8 T cells stimulated with anti-TCR-β mAb plus anti-CD28 mAb for 24 h were determined by immunoblotting. The protein amount of β-actin was used as a loading control. The numbers below the bands indicate the densitometry ratio with WT. The results of the immunoblot analyses are representative of at least three independent experiments with similar results and are presented as cropped images. The full-length blots are presented in Supplementary Figure 18.

Supplementary Figure 2

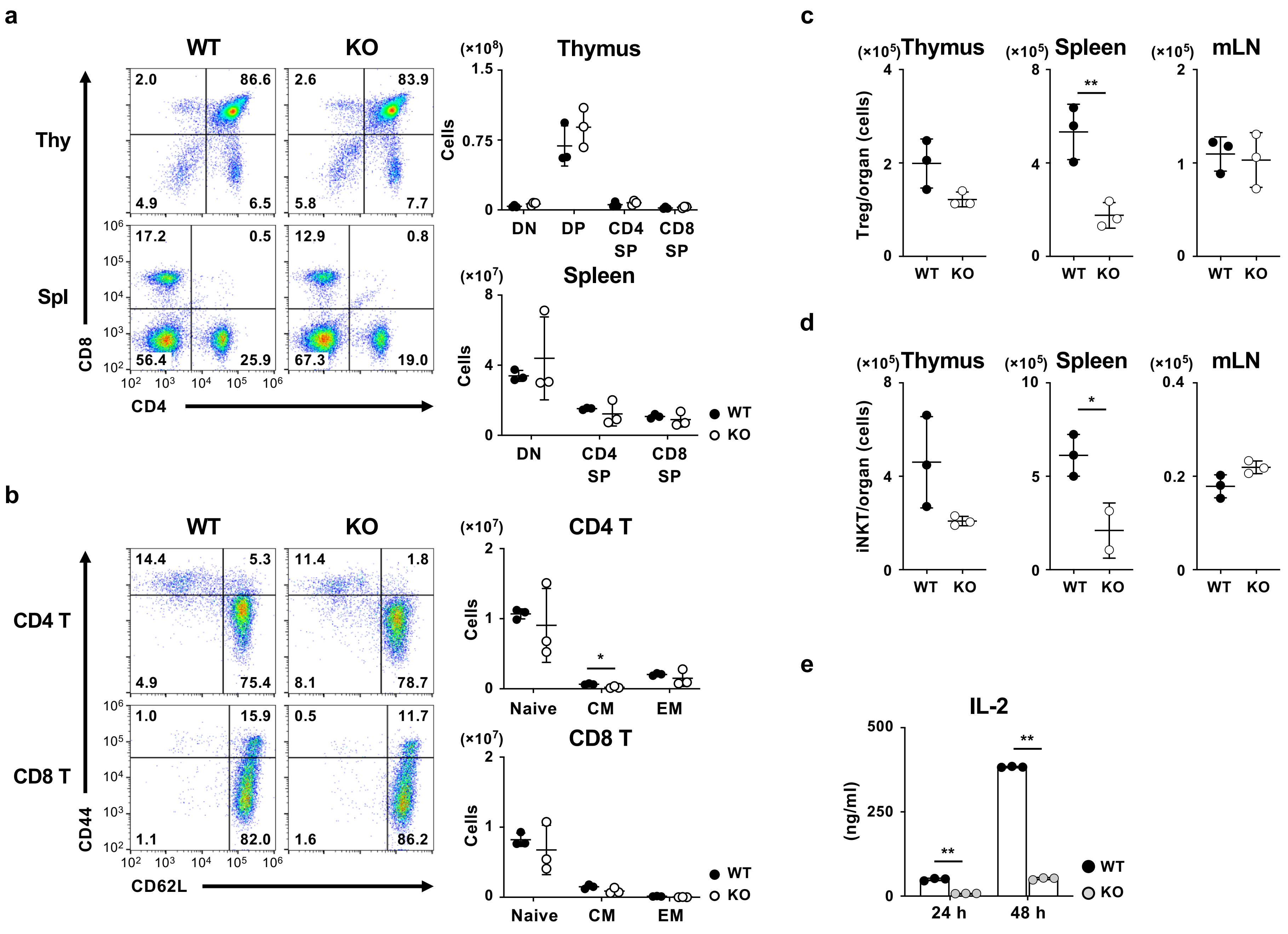

Supplementary Figure 2. Phenotypic characterization of *Pgam1* KO T cells. (a) Representative results of FACS analyses of the thymocytes and splenocytes in WT and *Pgam1* KO mice (left). The numbers of CD4^{neg} CD8^{neg} (DN), CD4^{pos} CD8^{pos} (DP), CD4^{pos} CD8^{neg} (CD4 SP) and CD4^{neg}CD8^{pos} (CD8 SP) cells are shown with the standard deviations (right) (n = 3, biological replicates). **(b)** Representative FACS profile of CD62L/CD44 in CD8 and CD4 T cells of the spleen from WT and *Pgam1* KO mice (left). The numbers of naïve (CD62L^{hi} CD44^{lo}), central memory (CM: CD62L^{hi}CD44^{hi}) and effector memory (EM: CD62L^{lo} CD44^{hi}) CD4 and CD8 T cells are shown with the standard deviations (right) (n = 3, biological replicates). **(c)** The numbers of Treg (Foxp3^{hi} CD25^{hi}) in the thymus, spleen and mesenteric lymph nodes (mLN) in WT and *Pgam1* KO mice were determined by FACS (n = 3, biological replicates). **(d)** The numbers of iNKT cells (CD1d-tet^{hi}) in the thymus, spleen, and mLN from WT and *Pgam1* KO mice were determined (n = 3, biological replicates). **(e)** The results of the ELISA for IL-2 in WT and *Pgam1* KO CD4 T cells stimulated with anti-TCR-β plus anti-CD28 mAbs for 24 and 48 h (n=3, biological replicates). The results are shown with the standard deviation. *P<0.05, **P<0.01 (Student's *t*-test).

Supplementary Figure 3

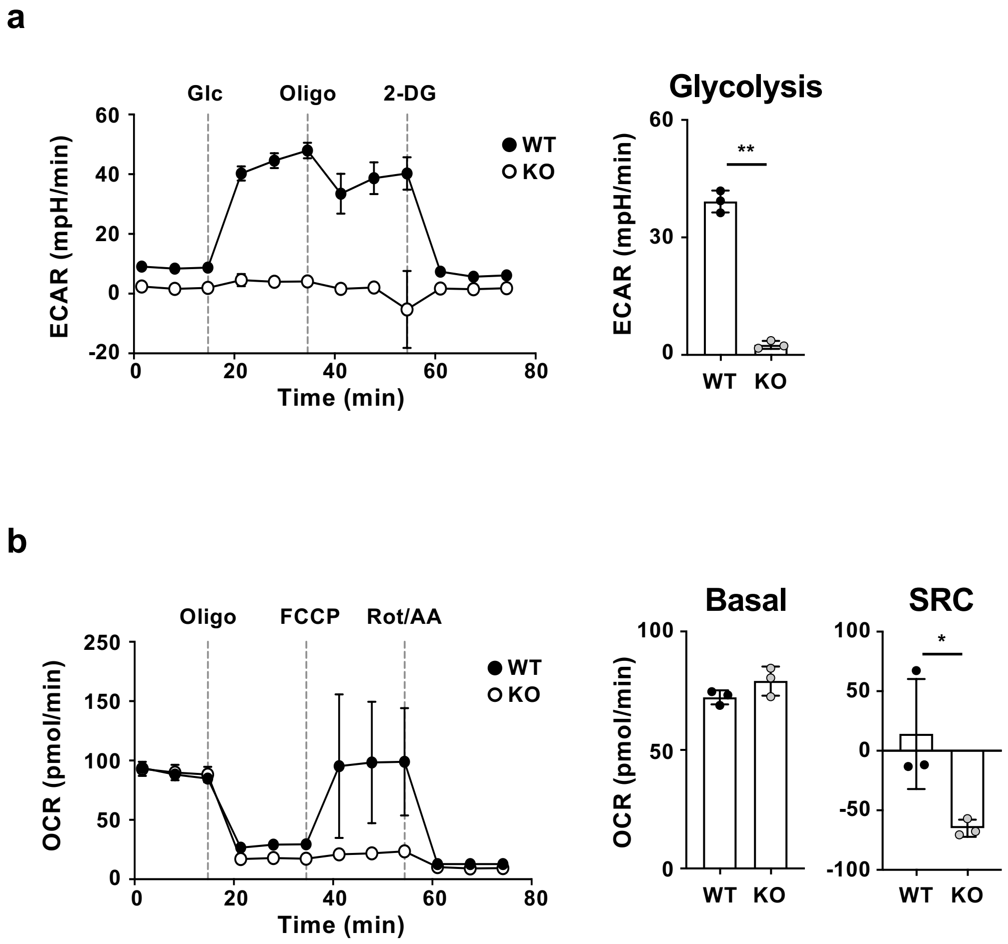

Supplementary Figure 3. Impaired glycolysis and mitochondrial respiration in *Pgam1* KO CD4 T cells. WT and *Pgam1* KO naïve CD4 T cells were stimulated with anti-TCR-β plus anti-CD28 mAbs for 36 h, and then the ECAR **(a)** and OCR **(b)** were determined. The error bars represent the standard deviation. *P<0.05, **P<0.01 (Student's *t*-test). WT and *Pgam1* KO naïve CD4 T cells were stimulated with anti-TCR-β plus anti-CD28 mAbs for 36 h, and then the ECAR **(a)** and OCR **(b)** were determined. The error bars represent the standard deviation. *P<0.05, **P<0.01 (Student's *t*-test).

Supplementary Figure 4

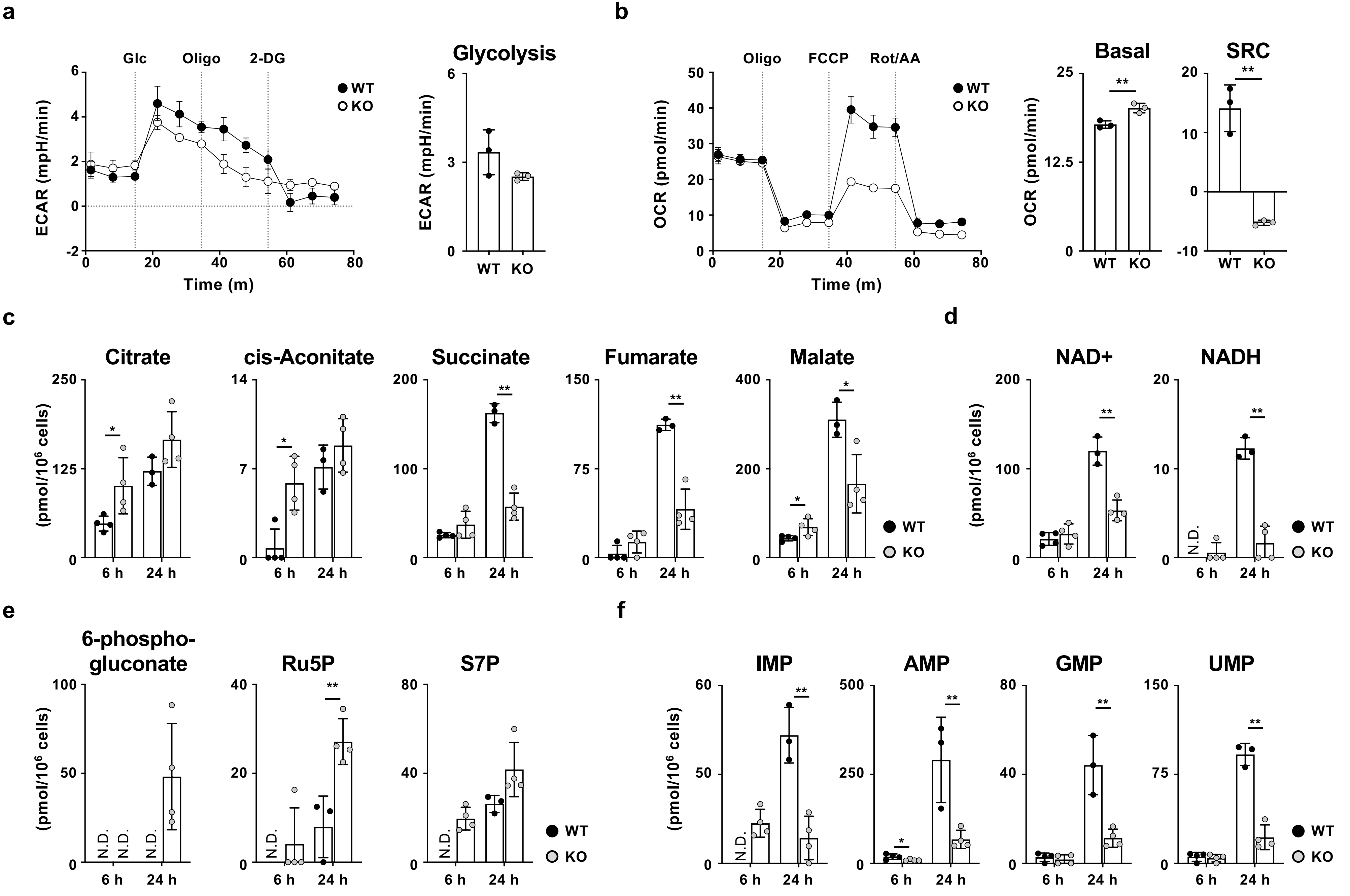

Supplementary Figure 4. WT and *Pgam1* KO naïve CD4 T cells were stimulated with anti-TCR-β plus anti-CD28 mAbs for 8 h, and then the ECAR **(a)** and OCR **(b)** were determined (n=3, biological replicates). **(c)** WT and *Pgam1* KO naïve CD8 T cells were stimulated with anti-TCR-β and anti-CD28 mAbs for 6 or 24 h, and the intracellular amounts of TCA cycle intermediates were determined (n=3-4, biological replicates). **(d)** The intracellular amounts of NAD^+ and NADH of the cells in (c). **(e)** The intracellular amounts of pentose phosphate pathway intermediates of the cells in (c). **(f)** The intracellular amounts of nucleotides of the cells in (c). Error bars represent the standard deviation. *P<0.05, **P<0.01 (Student's *t*-test).

Supplementary Figure 5

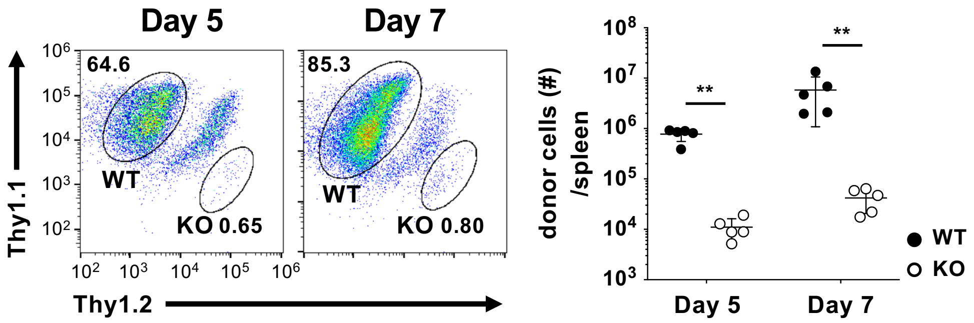

Supplementary Figure 5. 1:1 mixture of WT OT-1 Tg naïve CD8 T (Thy1.1$^+$)/*Pgam1* KO OT-1 Tg naïve CD8 T (Thy1.2$^+$) cells was adoptively transferred into WT congenic (Thy1.1$^+$Thy1.2$^+$) mice. The recipient mice were then infected with *Lm*-OVA to activate donor cells. The number of OVA-specific donor cells in the spleen was analyzed on days 5 and 7 after *Lm*-OVA infection. Representative FACS profiles (left) and the absolute number of donor cells in the spleen are shown with the standard deviations (right). Each point represents an individual mouse.

Supplementary Figure 6

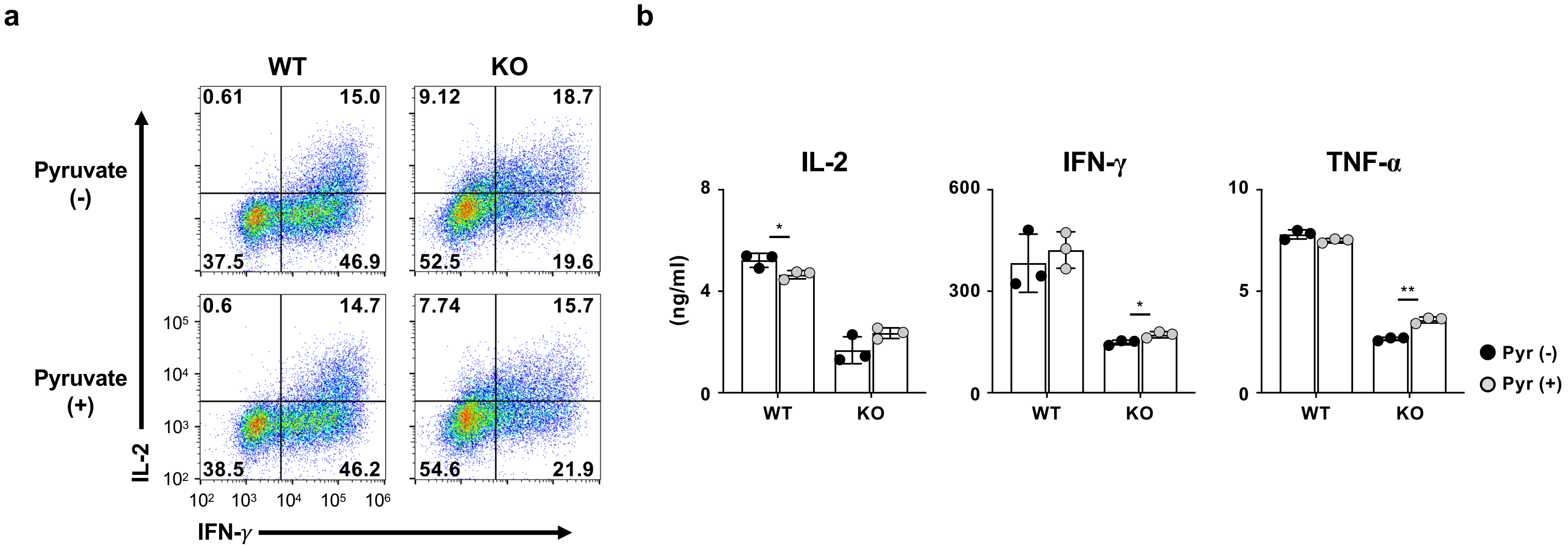

Supplementary Figure 6. The administration of pyruvate fails to restore effector CD8 T cell differentiation in *Pgam1* KO naïve CD8 T cells. (a) Representative results of the intracellular FACS analysis of IFN-γ/IL-2 in the WT and *Pgam1* KO CD8 T cells cultured under IL-2 conditions in the presence or absence of pyruvate for 5 days. The percentages of cells are indicated in each quadrant. The results of the FACS analyses are representative of at least three independent experiments with similar results. **(b)** The results of an ELISA for IL-2, IFN-γ and TNF-α in the supernatants of the cells in (a) (n=3, biological replicate). *P<0.05, **P<0.01 (Student's *t*-test).

Supplementary Figure 7

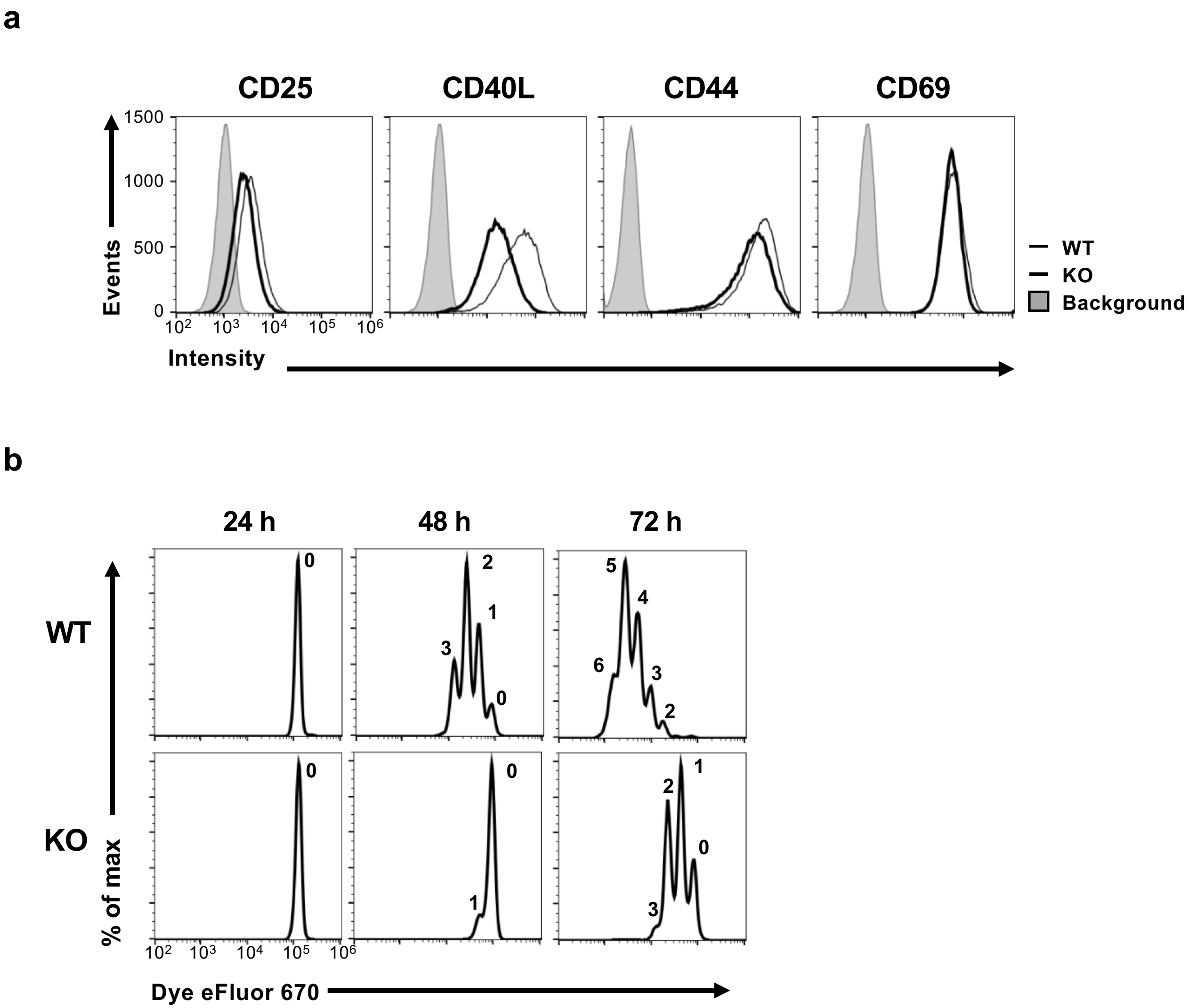

Supplementary Figure 7. (a) Representative results of FACS analyses of CD25, CD40L, CD44 and CD69 in WT and *Pgam1* KO CD4 T cells stimulated with anti-TCR-β mAb plus anti-CD28 mAb for 36 h. **(b)** WT and *Pgam1* KO naïve CD4 T cells were labeled with eFluor670 and stimulated with anti-TCR-β mAb plus anti-CD28 mAb. Cell division was detected by flow cytometry at 24, 48 and 72 h. The results of the FACS analyses are representative of at least three independent experiments with similar results.

Supplementary Figure 8

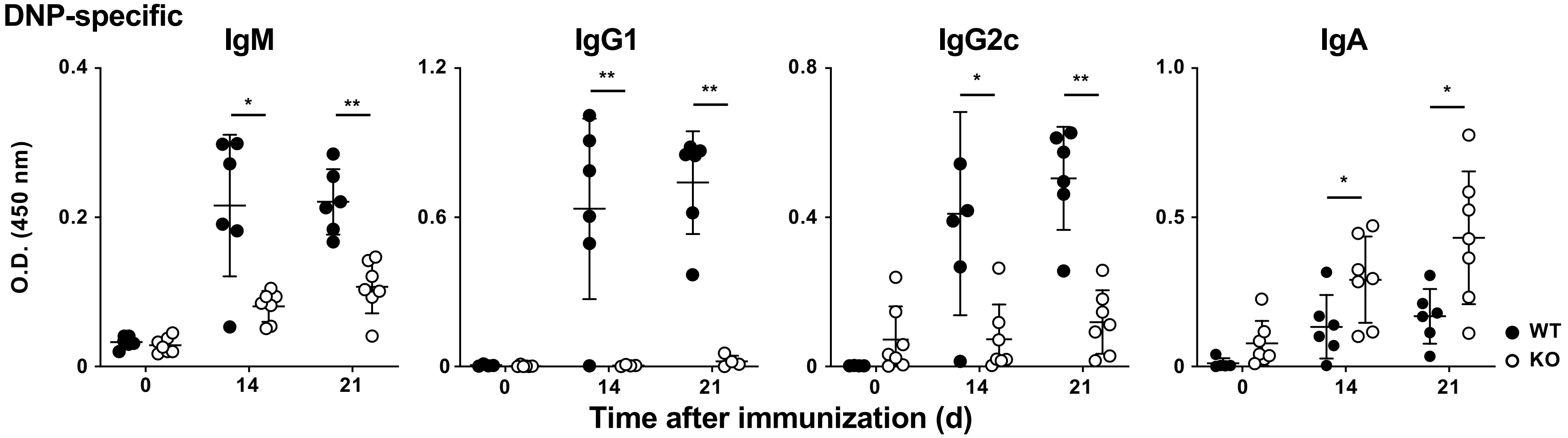

Supplementary Figure 8. WT and *Pgam1* KO mice were intraperitoneally immunized with 2,4-dinitrophenylated ovalbumin (DNP-OVA) in CFA on day 0, and then the mice were boosted with DNP-OVA in CFA on day 14. The serum was obtained on days 0, 14 and 21 and the amount of DNP-specific immunoglobulin were determined by an ELISA (n=6-7 per group). The results are indicated with the standard deviation. $*P<0.05$, $**P<0.01$ (Student's *t*-test).

Supplementary Figure 9

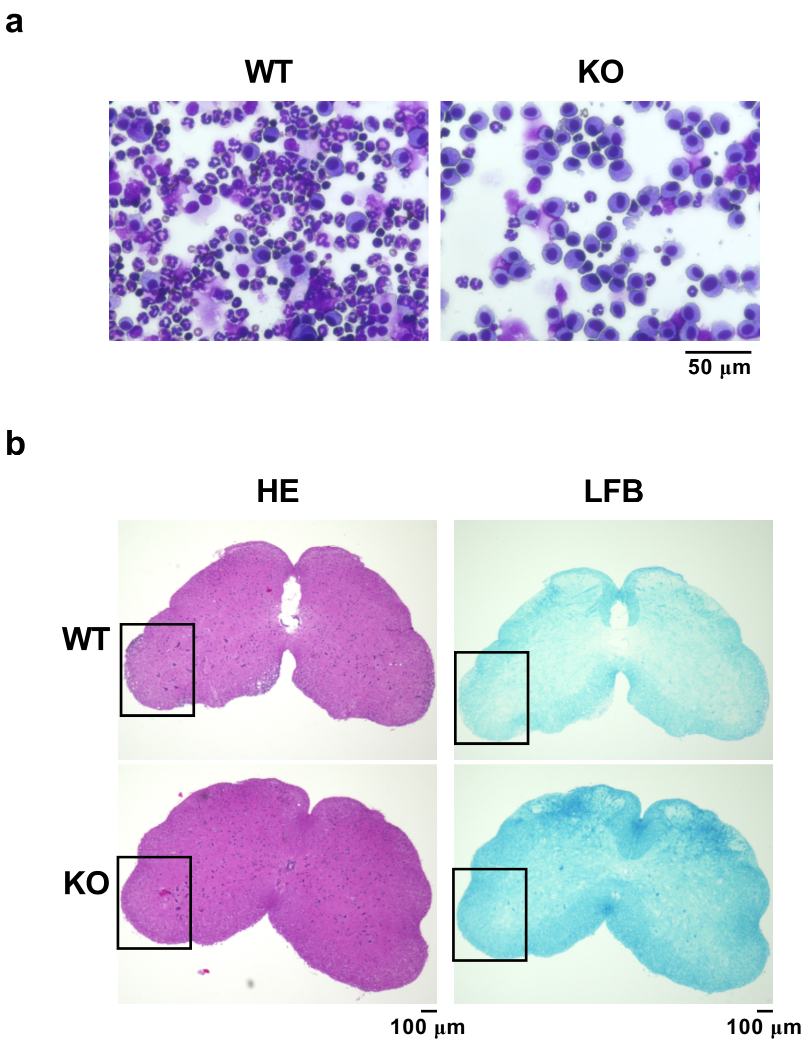

Supplementary Figure 9. (a) Diff-Quick staining of the BAL fluid cells derived from WT control and *Pgam1* KO mice with allergic inflammation is shown (Scale bars = 50 μm). **(b)** H&E (left) or LFB (right panels) staining of the spinal cords from WT and *Pgam1* KO mice with EAE (Scale bars = 100 μm). The squares show the sections presented with magnification in Figure 4f.

Supplementary Figure 10

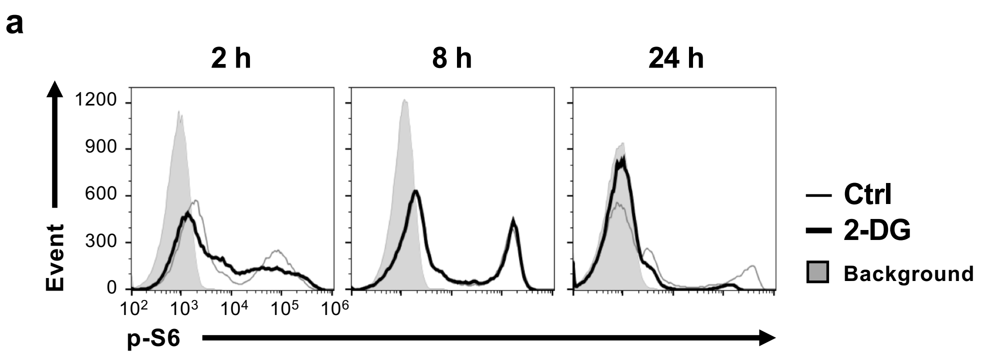

Supplementary Figure 10. Representative results of the intracellular FACS analysis of phospho-S6 (Ser235/236) in CD8 T cells stimulated in the presence or absence of 10 mM 2-DG for the indicated hours. The results of the FACS analyses are representative of at least three independent experiments with similar results.

Supplementary Figure 11

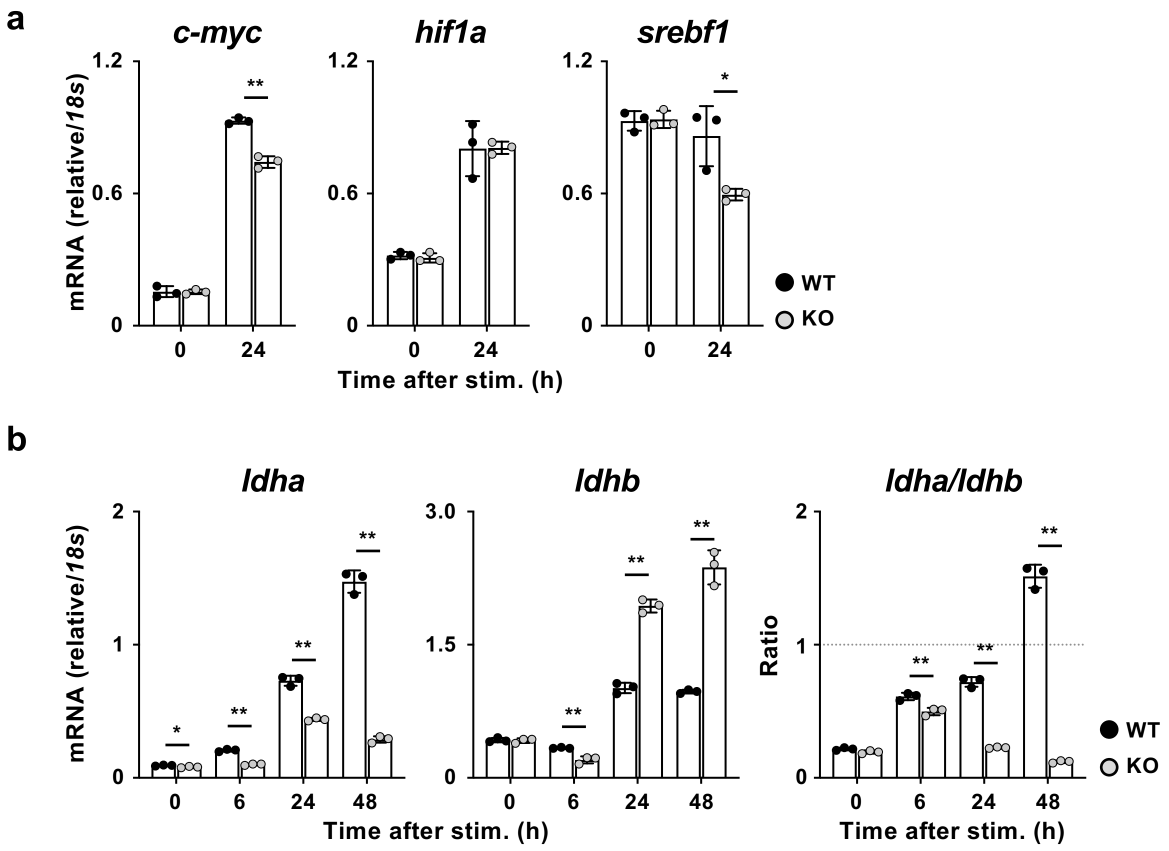

Supplementary Figure 11. (a) The results of the quantitative RT-PCR analysis of the *c-myc, hif1a* and *srebp1* mRNA in WT and *Pgam1* KO CD8 T cells. The results are presented relative to the expression of *18s* rRNA with the standard deviations (n = 3, technical replicates). **(b)** The results of the quantitative RT-PCR analysis of the *ldha, ldhb* mRNA (left and middle). The *ldha*/*ldhb* ratio in WT and *Pgam1* KO CD8 T cells was also determined (right). The results are presented relative to the expression of *18s* rRNA with the standard deviations (n = 3, technical replicates). *P<0.05, **P<0.01 (Student's *t*-test).

Supplementary Figure 12

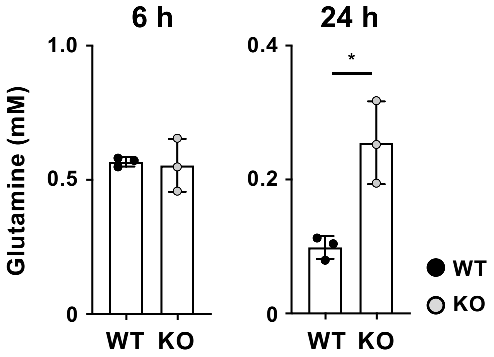

Supplementary Figure 12. WT and *Pgam1* KO naïve CD8 T cells were stimulated with anti-TCR-β and anti-CD28 mAbs for 6 or 24 h, and the glutamine concentration in the culture media was determined (n=3, biological replicates). The error bars represent the standard deviation. *P<0.05 (Student's *t*-test).

Supplementary Figure 13

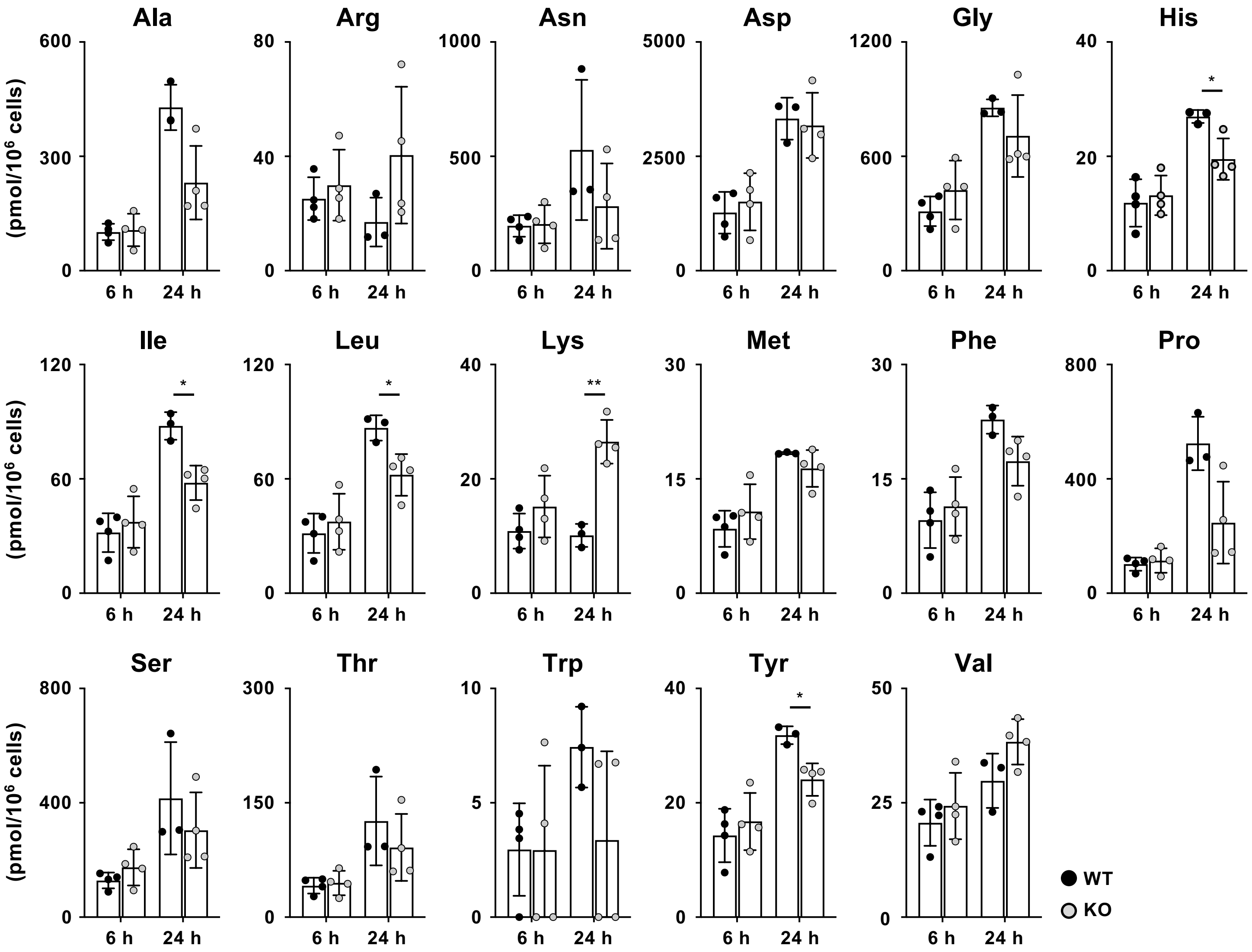

Supplementary Figure 13. The intracellular amino acid concentrations in activated WT and *Pgam1* KO CD8 T cells. WT and *Pgam1* KO naïve CD8 T cells were stimulated with anti-TCR-β and anti-CD28 mAbs for 6 or 24 h, and the intracellular amounts of amino acids were determined (n=3-4, biological replicates). The error bars represent the standard deviation. *P<0.05, **P<0.01 (Student's *t*-test).

Supplementary Figure 14

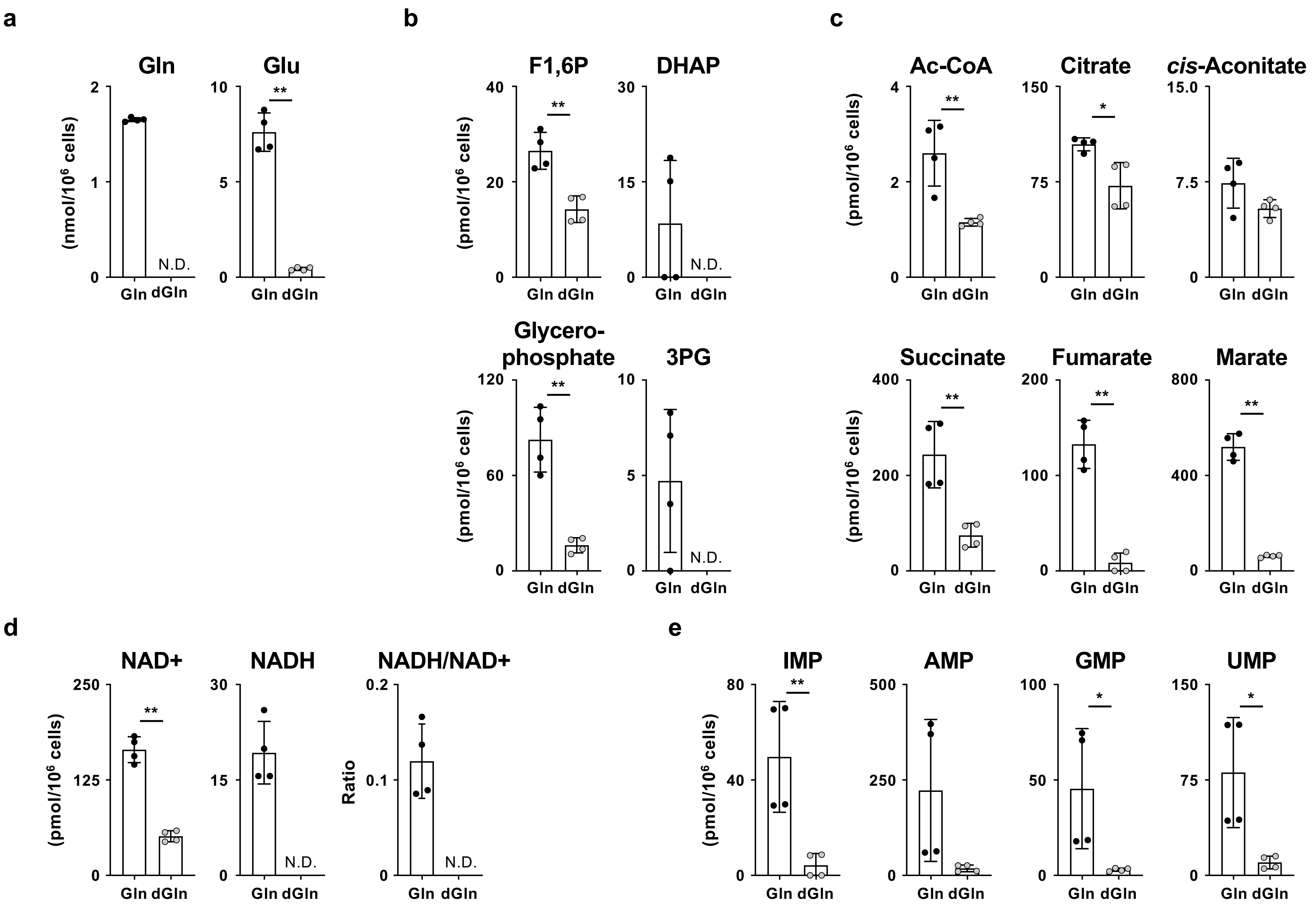

Supplementary Figure 14. (a) Naïve CD8 T cells were stimulated with anti-TCR-β and anti-CD28 mAbs in the presence or absence of glutamine for 24 h, and the intracellular amounts of glutamine and glutamate were determined (n=4, biological replicates). **(b)** The intracellular amounts of glycolytic intermediates of the cells in (a). **(c)** The intracellular amounts of TCA cycle intermediates of the cells in (a). **(d)** The intracellular amounts of NAD^+ and NADH of the cells in (a). The $NADH/NAD^+$ ratio was also determined. **(e)** The intracellular amounts of nucleic acids of the cells in (a). The results are shown with the standard deviation. *$P<0.05$, **$P<0.01$ (Student's *t*-test).

Supplementary Figure 15

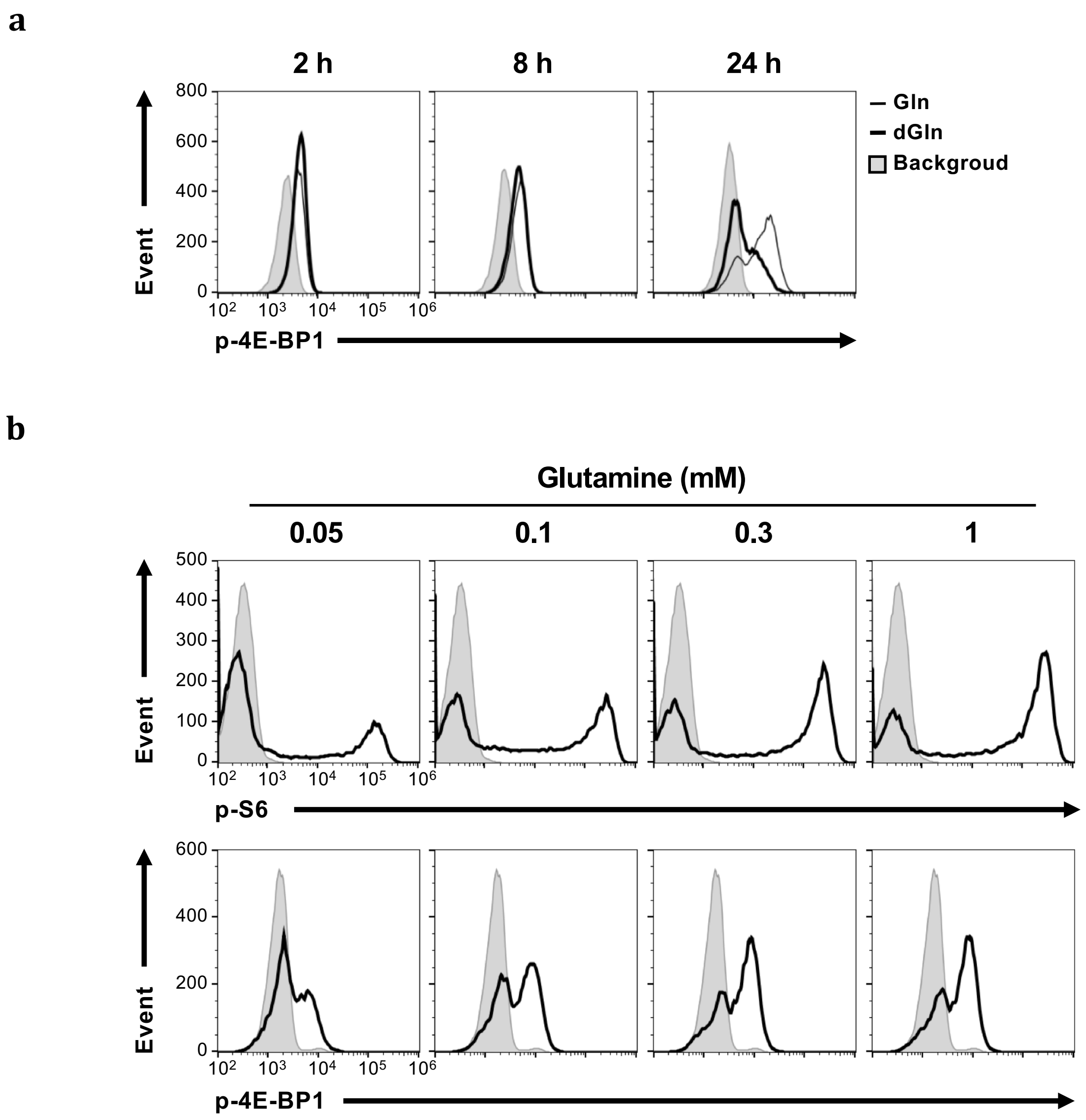

Supplementary Figure 15. (a) Representative results of intracellular FACS analyses of phospho-4E-BP1 (Thr37/46) in CD8 T cells stimulated with anti-TCR-β and anti-CD28 mAbs in the presence or absence of glutamine for the indicated number of hours. The results of the FACS analyses are representative of at least three independent experiments with similar results. **(b)** Representative results of intracellular FACS analyses of phospho-S6(Ser235/236) and 4E-BP1 (Thr37/46) of CD8 T cells cultured with the indicated concentrations of glutamine for 24 h. The results of the FACS analyses are representative of at least three independent experiments with similar results.

Supplementary Figure 16

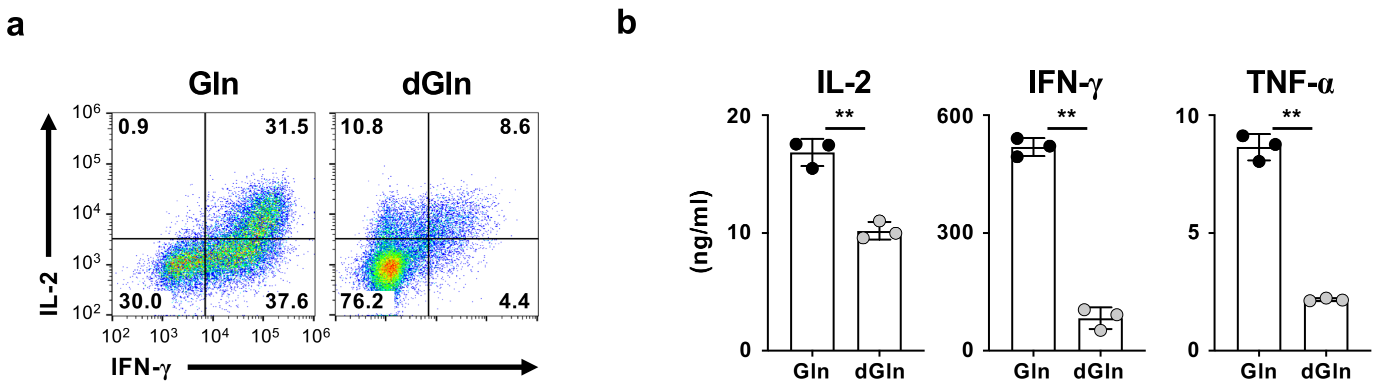

Supplementary Figure 16. (a) Representative results of intracellular FACS analyses of IFN-γ/IL-2 in effector CD8 T cells cultured under glutamine-sufficient or glutamine-depleted conditions. The results of the FACS analyses are representative of at least three independent experiments with similar results. **(b)** The results of an ELISA for IL-2, IFN-γ and TNF-α in the supernatants of the cells in (a) (n=3, biological replicate). The results are shown with the standard deviation. *$P<0.05$, **$P<0.01$ (Student's *t*-test).

Supplementary Figure 17

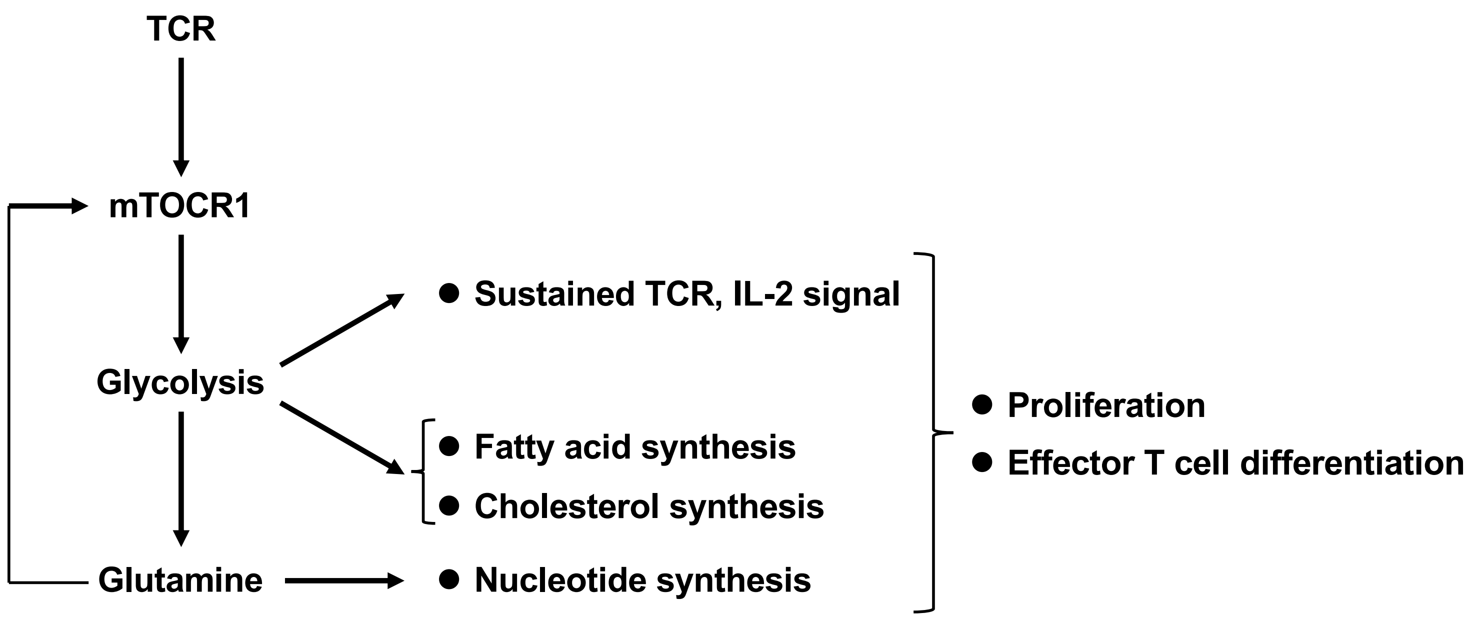

Supplementary Figure 17. Glycolysis, mTORC1 and glutamine cooperate to induce T-cell proliferation, differentiation and the T cell-dependent immune response. Glutamine sustains the mTORC1-, TCR-, IL-2-mediated signals and acts as a hub of metabolism in activated T cells.

Supplementary Figure 18

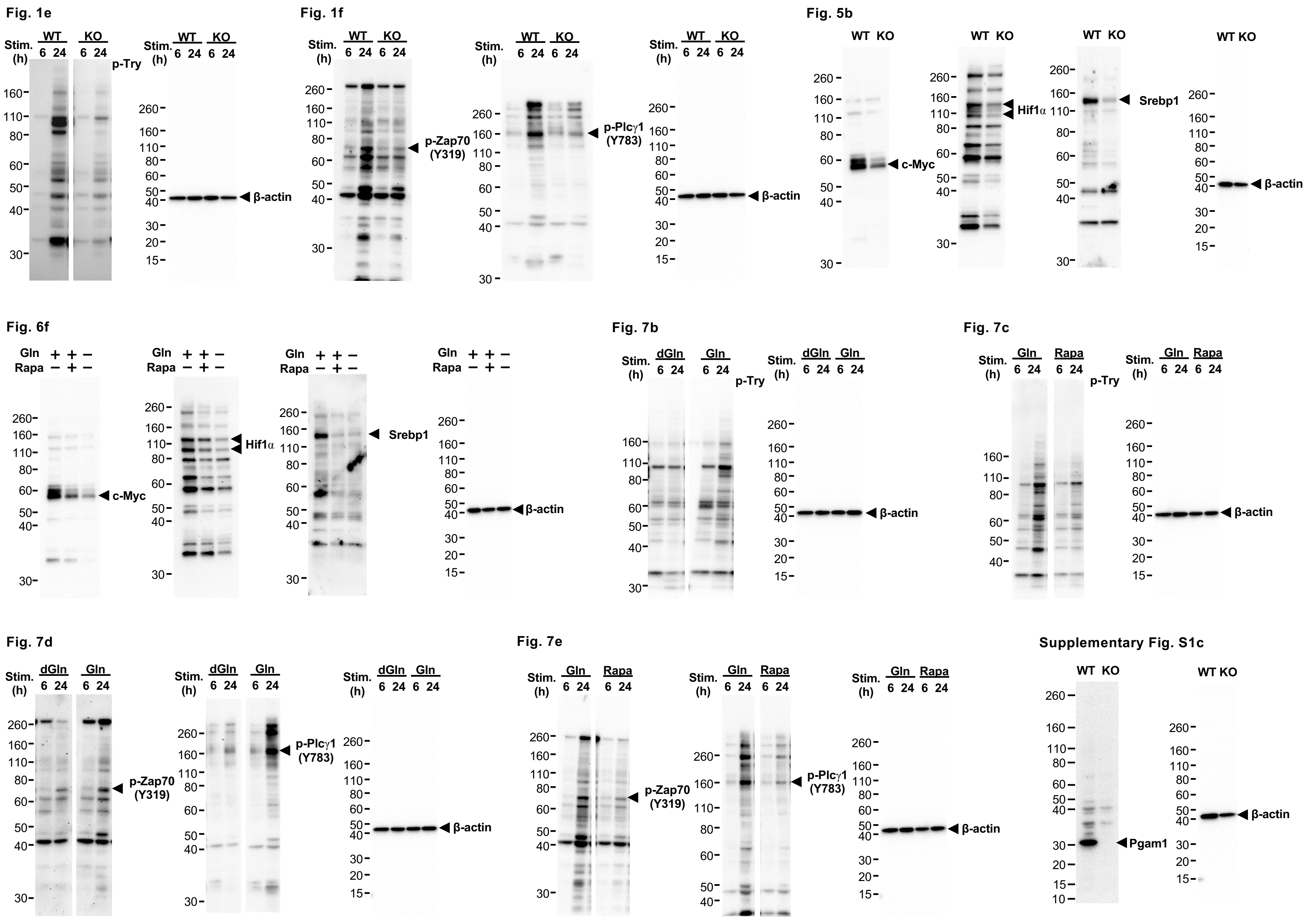

Supplementary Figure 18. Full size immunoblots of cropped blots in the main manuscript figures.

Supplementary methods

Measurement of DNP-specific immunoglobulins

WT and T cell-specific *Pgam1* KO mice were immunized intraperitoneally with 100 μg dinitrophenyl (DNP)-OVA (cat#DNP55-N-10; Alpha Diagnostics International, San Antonio, TX, USA) and Freund's complete adjuvant (CFA; cat#010-09543; Wako Chemicals). The immunized mice were intraperitoneally challenged with DNP-OVA and CFA on day 14 after immunization. The mice sera were obtained before immunization, on day 14 before challenge and on day 17. The levels of IgM, IgG1, IgG2c and IgA in the sera were determined using an ELISA.

Antibodies

The antibodies for the ELISA were as follows: anti-mouse IgM antibody (cat# A90-101P; Bethyl), anti-mouse IgG1 antibody (cat# A90-105P; Bethyl), anti-mouse IgG2c antibody (cat# A90-136P; Bethyl) and anti-mouse IgA antibody (cat# A90-103P; Bethyl).
