# Supplementary material for: T cell-specific deletion of Pgam1 reveals a critical role for glycolysis in T cell responses
Source: Commun Biol. 2020 Jul 24;3:394. doi: 10.1038/s42003-020-01122-w (PMC7382475; doi:10.1038/s42003-020-01122-w)
Supplement: Supplementary file 2 — Description of Additional Supplementary Files [file 42003_2020_1122_MOESM2_ESM.docx]

**Description of Additional Supplementary Files**

**File Name: Supplementary Data 1**

**Description:** Source data
