# Supplementary material for: T cell-specific deletion of Pgam1 reveals a critical role for glycolysis in T cell responses
Source: Commun Biol. 2020 Jul 24;3:394. doi: 10.1038/s42003-020-01122-w (PMC7382475; doi:10.1038/s42003-020-01122-w)
Supplement: Supplementary file 4 — Reporting Summary [file 42003_2020_1122_MOESM4_ESM.pdf]

nature research

Corresponding author(s): Masakatsu Yamashita
Last updated by author(s): Jul 3, 2020

Reporting Summary

Nature Research wishes to improve the reproducibility of the work that we publish. This form provides structure for consistency and transparency in reporting. For further information on Nature Research policies, see our Editorial Policies and the Editorial Policy Checklist.

Statistics

For all statistical analyses, confirm that the following items are present in the figure legend, table legend, main text, or Methods section.

n/a	Confirmed	
☐	☒	The exact sample size (*n*) for each experimental group/condition, given as a discrete number and unit of measurement
☐	☒	A statement on whether measurements were taken from distinct samples or whether the same sample was measured repeatedly
☐	☒	The statistical test(s) used AND whether they are one- or two-sided<br>*Only common tests should be described solely by name; describe more complex techniques in the Methods section.*
☒	☐	A description of all covariates tested
☒	☐	A description of any assumptions or corrections, such as tests of normality and adjustment for multiple comparisons
☐	☒	A full description of the statistical parameters including central tendency (e.g. means) or other basic estimates (e.g. regression coefficient) AND variation (e.g. standard deviation) or associated estimates of uncertainty (e.g. confidence intervals)
☐	☒	For null hypothesis testing, the test statistic (e.g. *F*, *t*, *r*) with confidence intervals, effect sizes, degrees of freedom and *P* value noted<br>*Give P values as exact values whenever suitable.*
☒	☐	For Bayesian analysis, information on the choice of priors and Markov chain Monte Carlo settings
☒	☐	For hierarchical and complex designs, identification of the appropriate level for tests and full reporting of outcomes
☒	☐	Estimates of effect sizes (e.g. Cohen's *d*, Pearson's *r*), indicating how they were calculated

Our web collection on statistics for biologists contains articles on many of the points above.

Software and code

Policy information about availability of computer code

Data collection	Data were collected using BECKMAN COULTER GALLIOS, Applied Biosystems Step One Plus and RIO RAD Chemi DOC XRS+
Data analysis	FlowJo, Image J and Excel

For manuscripts utilizing custom algorithms or software that are central to the research but not yet described in published literature, software must be made available to editors and reviewers. We strongly encourage code deposition in a community repository (e.g. GitHub). See the Nature Research guidelines for submitting code & software for further information.

Data

Policy information about availability of data

All manuscripts must include a data availability statement. This statement should provide the following information, where applicable:

- Accession codes, unique identifiers, or web links for publicly available datasets
- A list of figures that have associated raw data
- A description of any restrictions on data availability

The data that support the findings of this study are available from the corresponding author upon reasonable request. The raw data of main figure panels 1a, b, c d. 2f, 3b, d, f, 5c, d, e, 6a, b, c, d, g, 7a and Supplementary figures panels 1a, 2c, d, e, 3a, b, 4a, b, c, d, e, f, 6b, 11a, b, 12,13, 14, 15b are available as Supplementary data.

Field-specific reporting

Please select the one below that is the best fit for your research. If you are not sure, read the appropriate sections before making your selection.

☒ Life sciences ☐ Behavioural & social sciences ☐ Ecological, evolutionary & environmental sciences

For a reference copy of the document with all sections, see nature.com/documents/nr-reporting-summary-flat.pdf

Life sciences study design

All studies must disclose on these points even when the disclosure is negative.

Sample size	No prior sample-size calculation was performed. Statistical analysis was performed on the collected data and the sample size was determined to be sufficient based on the size and consistency of the difference between groups.
Data exclusions	No data were excluded from the analysis.
Replication	All attempts at replication was successful.
Randomization	All cells and animals were randomized among the groups.
Blinding	Investigators were generally not blinded. All parameters examined were quantifiable; hence, no risk of subconscious bias.

Reporting for specific materials, systems and methods

We require information from authors about some types of materials, experimental systems and methods used in many studies. Here, indicate whether each material, system or method listed is relevant to your study. If you are not sure if a list item applies to your research, read the appropriate section before selecting a response.

Materials & experimental systems

n/a	Involved in the study	
☐	☒	Antibodies
☒	☐	Eukaryotic cell lines
☒	☐	Palaeontology and archaeology
☐	☒	Animals and other organisms
☒	☐	Human research participants
☒	☐	Clinical data
☒	☐	Dual use research of concern

Methods

n/a	Involved in the study	
☒	☐	ChIP-seq
☐	☒	Flow cytometry
☒	☐	MRI-based neuroimaging

Antibodies

Antibodies used	Antibodies used in the current study have been listed in the Methods section and the Supplementary information.
Validation	All the antibodies have validation statements on the vendors websites.

Animals and other organisms

Policy information about studies involving animals; ARRIVE guidelines recommended for reporting animal research

Laboratory animals	Laboratory animals used in the current study have been listed in the Methods section.
Wild animals	Not applicable.
Field-collected samples	Not applicable.
Ethics oversight	Ethics oversight in the current study have been described in the Methods section.

Note that full information on the approval of the study protocol must also be provided in the manuscript.

Flow Cytometry

Plots

Confirm that:

☒ The axis labels state the marker and fluorochrome used (e.g. CD4-FITC).

☒ The axis scales are clearly visible. Include numbers along axes only for bottom left plot of group (a 'group' is an analysis of identical markers).

☒ All plots are contour plots with outliers or pseudocolor plots.

☒ A numerical value for number of cells or percentage (with statistics) is provided.

Methodology

Sample preparation	The samples were prepared according to the standard protocol from the distributors.
Instrument	BECKMAN COULTER GALLIOS
Software	FlowJo software
Cell population abundance	10,000 to 100,000 events were collected in predetermined gates for each group.
Gating strategy	Gates and quadrants were set based on isotype control staining.

☒ Tick this box to confirm that a figure exemplifying the gating strategy is provided in the Supplementary Information.
